# Tumor-Derived Exosomes Mediate the Instability of Cadherins and Promote Tumor Progression

**DOI:** 10.3390/ijms20153652

**Published:** 2019-07-26

**Authors:** Bowen Wang, Zengqi Tan, Feng Guan

**Affiliations:** Provincial Key Laboratory of Biotechnology, Joint International Research Laboratory of Glycobiology and Medicinal Chemistry, College of Life Science, Northwest University, Xi’an 710069, China

**Keywords:** cadherins, exosomes, epithelial–mesenchymal transition, microRNAs, long non-coding RNAs, tumor development

## Abstract

Cadherins, including E-cadherin, N-cadherin, VE-cadherin, etc., are important adhesion molecules mediating intercellular junctions. The abnormal expression of cadherins is often associated with tumor development and progression. Epithelial–mesenchymal transition (EMT) is the most important step in the metastasis cascade and is accompanied by altered expression of cadherins. Recent studies reveal that as a cargo for intercellular communication, exosomes—one type of extracellular vesicles that can be secreted by tumor cells—are involved in a variety of physiological and pathological processes, especially in tumor metastasis. Tumor-derived exosomes play a crucial role in mediating the cadherin instability in recipient cells by transferring bioactive molecules (oncogenic microRNAs (miRNAs) and long non-coding RNAs (lncRNAs), EMT-related proteins, and others), modulating their local and distant microenvironment, and facilitating cancer metastasis. In turn, aberrant expression of cadherins in carcinoma cells can also affect the biogenesis and release of exosomes. Therefore, we summarize the current research on the crosstalk between tumor-derived exosomes and aberrant cadherin signals to reveal the unique role of exosomes in cancer progression.

## 1. Introduction of Exosomes

Extracellular vesicles (EVs) are nanoscale vesicles secreted by cells, including apoptotic bodies, exosomes, and microvesicles. Exosomes are small EVs (sEVs) with a cup-shaped morphology and diameters between 40 and 150 nm [1]. The differences between exosomes and other EVs have been comprehensively revealed. Recently, researchers used high-resolution density gradient centrifugation and immune affinity capture to isolate highly purified exosomes, which showed that the DNA is absent in sEVs, and exosomes do not contain glycolytic enzymes, nor do they contain cytoskeletal proteins [2].

**Figure 1:**
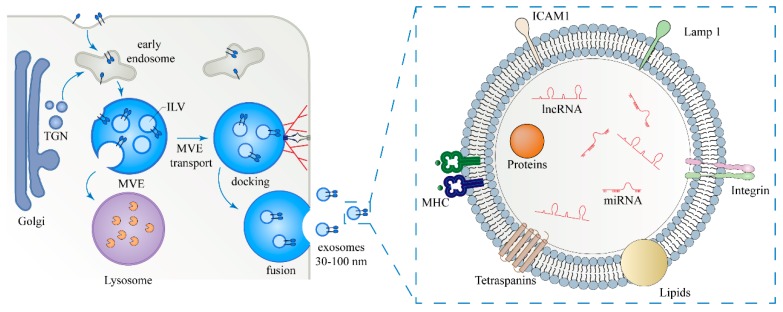
Biogenesis, secretion, and cargos of exosomes. Exosomes are formed as intraluminal vesicles (ILVs) by endocytosis at the plasma membrane (PM), budding into early endosomes and multivesicular body (MVB). Cargos, which are targeted to MVEs, originate from endocytosis at the PM or from the early endosomes via the trans-Golgi network (TGN). The fate of MVBs can be either fusion with lysosomes or docking and fusion with the plasma membrane, which allows the release of exosomes to the extracellular milieu. Exosome cargo contains non-coding RNAs (including microRNAs and lncRNAs), proteins, and lipids. TGN: trans-Golgi network; ICAM-1: intercellular cell adhesion molecule-1; and MHC: major histocompatibility complex.

The biogenesis pathway of exosomes has been widely studied. It is well accepted that exosomes are derived from the early endosome formed by budding inwards through the plasma membrane. Then, with help from the endosomal sorting complex required for transport (ESCRT), the transition of exosomes from early endosomes to late endosomes, known as multivesicular endosomes (MVEs), is completed. ESCRT consists of ~30 proteins that form four complexes including ESCRT-0, I, II, and III, and is critical for the release of exosomes. MVEs have two destinies: one is to enter lysosome and be degraded; the other is to fuse with the plasma membrane and release intraluminal vesicles (ILVs), i.e., exosomes, into extracellular environment [3,4] (Figure 1). Although it is known that MVEs have different destinies, the molecular mechanisms by which MVEs are sorted remain unclear.

Exosomes are found in cell culture supernatants and in different biological fluids, including blood, urine, breast milk, amniotic fluid, malignant ascites, etc. [5,6,7]. Exosomes are widely distributed, but how to get high quality exosomes has become another thorny problem. Exosome extraction methods include ultracentrifugation, density gradient centrifugation, immunocapture, etc., and each method has its pros and cons [8]. Ultracentrifugation has been most widely used for exosome extraction by virtue of its simplicity and rapidity, but purity of exosomes obtained by this method is not ideal enough. With the deepening cognition of exosomes, it is considered that exosomes with higher purity can better reveal their functions. Compared to ultracentrifugation, density gradient centrifugation has the advantage of obtaining exosomes with higher purity, but its disadvantage is that it takes more time and energy, and few exosomes are isolated [9]. Using magnetic beads which conjugated with specific antibodies to capture exosomes is another better strategy, but this method is expensive and produces few exosomes [10]. After isolation, purified exosomes need to be further identified. Currently, exosomes are identified by three complementary methods: exosomes morphology is observed by electron microscope, the particle size is detected by nanoparticle tracer analysis (NTA), and exosomal markers including CD81, CD63, and CD9, etc., are detected by Western blot [2,11].

Like cells, exosomes have the structure of phospholipid bilayer, which can better protect the enveloped materials. Exosomes are encapsulated by proteins and lipids, and contain functional proteins and genetic material, such as mRNAs, microRNAs (miRNAs), and long non-coding RNAs (lncRNAs). An exosome’s source determines the difference in exosomal composition, which reflects the physiological status and functionality of the host cells [12,13,14] (Figure 1). Initially, exosomes were thought to be “trash cans” that transported cellular waste into the extracellular environment and were then metabolized with the circulatory system. Later, it was found that exosomes have a greater role in transferring proteins, lipids, and nucleic acids from host cells to recipient cells [15]. Exosomes are involved in many biological functions including antigen-presentation, cell-cell communication, immunomodulation, microenvironment remodeling, and tumor metastasis [16,17,18]. Usually, patients with different types of cancer release exosomes which contain different signatures of miRNAs into the circulation, which can be measured as diagnostic biomarkers [19].

Current research has shown that abnormalities in cadherin signals are strongly associated with exosomes, especially those derived from tumors [20,21]. Therefore, we summarize recent studies on the instability of cadherins and tumor progression mediated by tumor-derived exosomes.

## 2. Key Role of Cadherins in EMT

Cadherins, a type of cell adhesion molecule, are a class of type-1 transmembrane proteins, and their functions are highly dependent on calcium (Ca^2+^) ions in a homophilic manner. The cadherin superfamily shares the extracellular cadherin repeats, a single transmembrane domain, and a cytoplasmic domain [22]. Cadherins play important roles in the formation of adhesion junctions and maintenance of the cell–cell adhesion. Typically, epithelial cells express E-cadherin, whereas mesenchymal cells express various cadherins, including N-cadherin, R-cadherin, and cadherin-11 [23]. Specifically, endothelial cells express VE-cadherin [24].

Cadherins often undergo significant changes in the epithelial–mesenchymal transition (EMT) process. Epithelial cells undergo morphological transformation to mesenchymal phenotype and acquire stronger migration ability accompanied by cadherin dysregulation, which is called “cadherin switch” [25]. “Cadherin switch” from E-cadherin to N-cadherin is a research hotspot because it is extremely important for understanding the mechanism of tumor metastasis [26]. Loss of E-cadherin expression is the hallmark of the EMT process. E-cadherin is widely expressed in most epithelial tissues. The mature E-cadherin contains a cytoplasmic domain, transmembrane domain, and ectodomain. The cytoplasmic domain of the E-cadherin is complexed with either β-catenin or γ-catenin (plakoglobin). β-catenin and γ-catenin bind directly to α-catenin, and form the distinct cadherin–catenin complexes [27]. Plakoglobin not only binds to E-cadherin to form adherens junctions, but also interacts with desmosomal cadherins, such as desmoglein, to form desmosomal junctions which are not associated with α-catenin [28]. This adherens junction complex is linked to the actin cytoskeleton, further mediating the mechanical stability [29]. p120 catenin is a member of the catenin family and can bind to the juxta membrane domain (JBD) of E-cadherin, but not to α-catenin at the other end [30]. The expression of E-cadherin is regulated by various transcription factors, such as Slug, Snail, etc., which bind to the E-box region of the E-cadherin promoter region to inhibit the expression of E-cadherin. Recent findings have reported that lysyl oxidase-like 2 (LOXL2) could act as a modulator for Snail, and synergize with another transcription factor, bHLH transcription factor E47, to reduce E-cadherin levels [31]. The loss of E-cadherin’s homophilic binding further results in the loss of cell–cell adhesion and cell polarity [32,33]. Meanwhile, the role of E-cadherin in intracellular and intercellular signal transduction has also been widely explored [34,35,36].

Gain of mesenchymal N-cadherin expression is observed in cells undergoing EMT, and serves as another important feature of EMT. Although high expression of N-cadherin is not considered to be a promoter of tumor growth, it does correlate with tumor progression [37,38]. Other types of cadherins, such as cadherin-6 and P-cadherin, have also attracted attention in recent years. Cadherin-6 was abnormally expressed in thyroid cancer. Further studies have demonstrated that the activation of cadherin-6 could promote autophagy and enhance EMT characteristics, demonstrating a new function of cadherins [39]. In nasopharyngeal carcinoma, Epstein-Barr viral (EBV) oncoprotein LMP1 inhibited the transcription of miR-203, which specifically targeted cadherin-6 by activating NF-κB signaling. Meanwhile, cadherin-6 was a hub that linked the NF-κB and TGF-β signaling pathways to promote the development of EMT and cancer metastasis [40].

It is well known that multiple signaling pathways are involved in EMT and affect the expression of cadherins such as Wnt, TGF-β, Notch, etc. [41]. β-catenin is a messenger in EMT, connecting signals in and out of the nucleus. In the classical Wnt signaling pathway, cytoplasmic β-catenin evades degradation, accumulates in the cytoplasm, and finally translocates to the nucleus. Therefore, β-catenin is involved in the induction of EMT in physiological processes and tumorigenesis progression. Under normal conditions, E-cadherin can recruit β-catenin to adherens junctions, retard the β-catenin into the nucleus, and inhibit β-catenin activation of target gene expression. In contrast, loss of E-cadherin destructs the cadherin–catenin complexes and allows β-catenin to localize to the nucleus and exert its transcriptional activity. It is evident that both β-catenin and p120 are required for transcriptional activation of target genes—not just one in some cases [42]. N-cadherin and Wnt signaling pathways also compete for cellular β-catenin. Although it is generally believed that the increased expression of N-cadherin is positively correlated with tumorigenesis, it has also been reported that overexpression of N-cadherin transfers β-catenin from the nucleus to the cytoplasm and locates β-catenin to the plasma membrane, which may inhibit Wnt signaling and tumor development. Therefore, the role of N-cadherin in tumorigenesis needs to be further revealed [43,44], and the role of N-cadherin in tumorigenesis needs to be further revealed.

TGF-β-inducing EMT is divided into the Smad-dependent and non Smad-dependent signaling pathways. In the Smad-dependent pathway, TGF-β binding to TGF-β type I and II receptors on the cell membrane phosphorylates Smad2/3 of the Smad protein family, and then the phosphorylated Samd2/3 forms a complex with Samd4 leading to nuclear translocation from the cytoplasm. Subsequently, the Smad complex transported into the nucleus mediates the expression of relevant target transcriptional factors, which could inhibit the expression of E-cadherin [45]. MicroRNAs induced by the Smad complex suppress E-cadherin and promote the expression of characteristic proteins in tumor cells to accelerate EMT [46]. However, some miRNAs perform the opposite function. The miR-200 family is the well-known miRNA molecule that regulates tumor EMT. Studies show that expression of the miR-200 family gene was down-regulated in the breast cancer EMT model induced by TGF-β, while overexpression of the miR-200 family could reverse EMT [47].

## 3. Exosomal miRNAs and Cadherins

**Figure 2:**
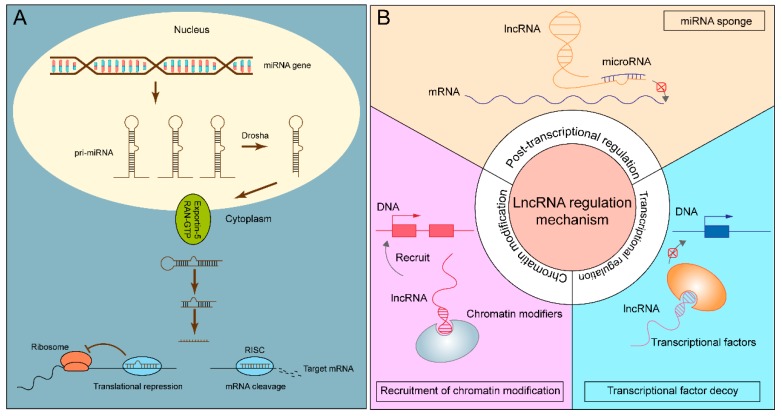
The role of miRNAs and lncRNAs. (**A**) The biogenesis and functional mechanism of miRNAs. (**B**) The regulatory aspects of lncRNAs.

MicroRNAs are small RNAs with a length of 18–25 nucleotides. In the canonical miRNA biogenesis pathway, genes responsible for coding are transcribed into pri-miRNA, which are cut into pre-miRNA with a stem-loop structure using Drosha-RNase [48]. Subsequently, pri-miRNA is transported from the nucleus to the cytoplasm with the help of Ran-GTP and Exportin-5. Dicer, a member of the RNase III family, specifically recognizes double-stranded RNA [49]. In Dicer-dependent miRNA processing, Dicer cuts pre-miRNA into 18–25 nucleotide double-stranded miRNA. After unwinding, one of the strands of double-stranded miRNA binds to Dicer and Argonaute to form RNA-induced silencing complex (RISC) that inhibits target gene transcription [50]. However, Studies have shown that miRNAs can be produced without Drosha or Dicer, which are known as the non-classical miRNA biogenesis pathway [51,52]. Exosomes-mediated secretion of human Ago proteins, other miRNA biogenesis machinery, and RISC components remains an unsettled issue. In 2014, pre-miRNAs, Dicer, AGO2, and TRBP were found in cancer exosomes, and pre-miRNA could even be processed into mature miRNA [53]. In 2016, McKenzie et al. showed that Ago2 existed in exosomes [54]. However, the latest reference in 2019 by Jeppesen et al. demonstrated that extracellular Ago proteins resulted from the contamination of crude sEVs samples with P-body, stress granules, or GW bodies, while other miRNA-associated proteins were absent from crude sEVs. In human plasma, Ago2 was present in the gradient-purified non-vesicle (NV) pool, but absent from the sEVs pool [2]. Typically, miRNAs target the 3’UTR (untranslated region) of mRNAs, but a few can also target 5′UTR and CDS (coding sequence) [55]. There are two main mechanisms of miRNAs that cause gene silencing. miRNAs associate with their mRNA targets by base-pair complementarity. The complete complementary pairing of miRNAs and mRNAs triggers mRNA degradation, while the incomplete complementary pairing of miRNAs and mRNAs inhibits protein synthesis or causes mRNA degradation (Figure 2A). Recent studies demonstrate that miRNAs play an essential role in cell cycle, circadian rhythms, tumor development and progression, etc. [56,57,58]. Moreover, circulating miRNAs derived from exosomes, a potential biomarker for cancer diagnosis and prognosis, are widely investigated in various cancers [59,60,61]. 

Exosomes carrying miRNAs have a significant impact on the expression of cadherins (Table 1). Usually, tumor cells express the increased oncogenic miRNAs, and the exosomes secreted by tumor cells could cargo these oncogenic miRNAs to recipient cells and modulate cadherin levels. For example, triple-negative breast cancer derived exosomal miR-9 could be transferred to normal fibroblasts (NFs). Exosomal miR-9 affects the status of NFs and promotes the cell malignant transformation from NFs to cancer-associated fibroblasts (CAFs), which further modify the tumor microenvironment and mediate intercellular communication. In addition, the co-culture of breast cancer cells with NFs transiently transfected with miR-9 demonstrated that miR-9 enhances tumor cells motility by reducing E-cadherin [62]. In order to enter the circulatory system and form metastatic sites in the distal tissue/organs, tumor cells will break through the endothelial barrier. Among the cadherins, vascular endothelial cadherin (VE-cadherin) is a vital adhesion molecule of endothelial cells and can be directly targeted by exosomal miR-103 and miR-939. The exosomal miR-103 secreted by hepatocellular carcinoma cells (HCCs) destroyed the adhesion junctions of endothelial cells by directly inhibiting the expression of VE-cadherin. The loss of adhesion between endothelial cells enhanced vascular permeability and was more conducive to cancer metastasis [6]. Exosomes secreted by breast cancer cells may carry miR-939 to reduce the expression of VE-cadherin and decrease cell-cell adhesion, which may explain the correlation between high miR-939 expression and low survival rates [63].

On the other hand, loss of certain tumor suppressor miRNAs in exosomes may induce the occurrence of EMT. DNA methyltransferases (DNMTs), a class of important molecules in epigenetics, are abnormally expressed in cancer [64]. The high level of DNA methylation modification caused by miRNA-deficiency regulates gene expression to promote tumor development. It was found that CAFs-derived exosomal miR-148b had a low abundance which partially promoted cancer metastasis, while the increase of miR-148b could inhibit DNMT1 translation in endometrial tumor cells accompanied by up-regulated E-cadherin and down-regulated N-cadherin [65]. It provided a new sight that the deletion of tumor suppressor miRNAs in exosomes induce EMT and mediate the instability of cadherin.

Notably, environmental stimulation can alter the composition of exosomes to facilitate tumor development. The tumor microenvironment is complex and diverse, with characteristics of hypoxia, low pH, and high pressure, which provide favorable conditions for tumor migration, invasion, and angiogenesis [66,67]. For example, the increased expression of miR-21 in exosomes secreted by hypoxic oral squamous cell carcinoma (OSCC) cells downregulated the expression level of E-cadherin [68]. Wang et al. demonstrated that miR-301a-3p was enriched in exosomes from hypoxic pancreatic cancer cells. Hypoxic exosomal miR-301a-3p induced macrophage polarization by activating the PTEN/PI3Kg signaling pathway. Polarized macrophages, in turn, promoted malignant transformation of pancreatic cancer cells, characterized by increased cell migration, invasion, and other EMT phenomenon [69].

A recent study showed that the expression of exosomal miRNA would be altered following the EMT process [70]. When treated with TGF-β1, human lung cancer cells A549 obtained a mesenchymal phenotype accompanied by a decrease in E-cadherin and the elevated expression of mesenchymal markers. At the same time, the miRNA profile of M-exosomes (derived from M-phenotype A549 cells) was obviously different compared with E-exosomes (derived from E-phenotype A549 cells). The results revealed that M-exosomal miRNAs were associated with malignant transformation and metastasis [70]. Similarly, an earlier study also demonstrated TGF-β1 could induce EMT in A549 cells resulting in the discrepant expression profile of exosomal proteins and miRNAs [71]. In addition to inducible factors, drug treatment also affects the expression of exosomal miRNA secreted by tumor cells. For example, exosomal expression levels of miR-200c and miR-141 were up-regulated by decitabine treatment in human colorectal cancer (CRC) cell lines [72].

## 4. Exosomal lncRNAs and Cadherins

lncRNAs are a class of RNA molecules with a length greater than 200 nt. Compared to miRNAs, lncRNAs are less conserved among species and more tissue-specific [73]. LncRNAs are transcribed from intergenic regions (large intergenic ncRNAs (lincRNAs)), intragenic regions, or specific chromosomal regions [74,75,76]. lncRNAs derived from intragenic regions can be classified in antisense, overlapping, intronic, and bidirectional orientations, which are related to protein-coding genes or gene regulatory regions. Furthermore, lncRNAs are processed after transcription, such as polyadenylation, alternative splicing, RNA editing, etc. [77]. The regulatory aspects of lncRNAs are divided into chromatin modification, transcriptional regulation, and post-transcriptional regulation. In other words, lncRNAs can interact with DNA, RNA, or protein to regulate gene expression [78,79,80] (Figure 2B). In terms of epigenetic regulation, lncRNA-*Xist* (X-inactive specific transcript) plays a typical role in X-chromosome inactivation. *Xist* is transcribed by the X chromosome and then wrapped around this chromosome. By recruiting DNA methylase and histone deacetylase, *Xist* leads to the suppression of gene expression on the overall level of X chromosome. *Tsix*, the antisense transcription product of *Xist*, effectively prevents the accumulation of *Xist*, thus maintaining the X-chromosome’s activity [81]. In addition, the transcription of lncRNAs may interfere with the transcription of neighboring protein-coding genes and degrading targeted mRNAs and acting as decoy for transcriptional regulators [82].

As the emerging regulators, lncRNAs have been shown to stimulate the tumor metastasis by mediating the expression of cadherins [83,84]. For example, high levels of lncRNA-ATB inhibited the function of miR-200 family, thereby enhancing the expression of transcriptional factor ZEB1 and promoting EMT in colon cancer [85]. In contrast, lncRNA-MEG3 can adsorb miR-421, which targets E-cadherin and further inhibits cell invasion [86]. More directly, TGF-β induced EMT and enhanced the level of lncRNA-HIT which accelerated tumor development by targeting E-cadherin in NMuMG cells [87]. In addition, multiple lncRNAs can interact with the histone-lysine N- methyltransferase enzyme EZH2 (enhancer of zeste homolog 2), which modifies histone methylation and inhibits E-cadherin transcription [88,89].

RNA sequencing analysis showed that certain lncRNAs including lncUEGC1 and LINC00477, were enriched in the tumor-derived exosomes and the expression profile of lncRNAs may be used to distinguish cancer patients from healthy controls [90,91]. Furthermore, lncRNAs were found to be implicated in EMT and have an impact on tumor development [92]. Xue et al. demonstrated that lncRNA-UCA1 expression was higher in hypoxic exosomes than normoxic exosomes, and hypoxic exosomal lncRNA-UCA1 promoted tumor development through EMT [93]. In addition, the exosomal lncRNA linc-ROR from thyroid cancer stem-like cells (CSCs) was necessary to induce EMT in normal thyroid cells [94]. Li et al. found that lncRNA-Sox2ot in exosomes was associated with pancreatic ductal adenocarcinoma progression. *Sox2* (SRY-related HMG-box gene 2), a transcriptional factor, has been shown to promote various tumors metastasis and drug resistance [95,96,97]. The miR-200 family not only directly targets *Sox2*, but also multiple transcriptional factors, such as ZEB1/2, to regulate EMT markers [98]. As competitive endogenous RNA (ceRNA), lncRNA-*Sox2*ot up-regulated *Sox2* and promoted EMT by competitively binding to the miR-200 family [99]. Unlike miRNA sponges, exosomal lnc-MMP2-2 can bind to the upstream site of MMP-2 gene, enhance the expression of MMP-2, and promote EMT [100]. In contrast, lncRNA NONHSAT105177 inhibited EMT by up-regulating E-cadherin, down-regulating various EMT inducible transcriptional factors and mesenchymal markers [101]. Although studies on exosomal lncRNAs-mediated the expression of cadherins are relatively few (Table 1), the above findings have laid a foundation for unraveling of the mystery of the association between exosomes and cadherin.

## 5. Exosomal Proteins and Cadherins

In addition to the aforementioned regulatory modalities of transferring RNAs, exosomes can also mediate the instability of cadherins by directly delivering certain proteins to recipient cells. Similar to nucleic acids in exosomes, proteomics analysis revealed that tumor-derived exosomes contain more oncogenic proteins, or perhaps less cancer suppressor proteins [102].

There is increasing evidence that many proteins are involved in the regulation of cadherins. It is known that the interaction of fibroblast growth factors (FGFs) with their receptors, fibroblast growth factor receptors (FGFRs), facilitates cancer progression [103]. Shi et al. found that FGF19 was highly expressed in exosomes derived from mesenchymal stem cells (MSCs). After treatment with MSC-exosomes, phosphorylated FGFR4 and ERK in nasopharyngeal carcinoma (NPC) cells increased, and the expression of EMT markers changed, presenting as down-regulation of E-cadherin, up-regulation of N-cadherin, and vimentin. It suggested that FGF19 carried by MSC-exosomes binds FGFR4 in NPC cells to activate the downstream ERK signaling pathway and thereby induce EMT [104]. Nakamura et al. demonstrated that exosomes from epithelial ovarian cancer (EOC) caused cells to more easily break through the mesothelial barrier. Further investigation revealed that high expression of CD44 in exosomes could lead to down-regulation of E-cadherin and up-regulation of MMP9, which degraded adhesive molecules in human peritoneal mesothelial cells [105]. Similarly, the epithelial growth factor receptor (EGFR)—a transmembrane glycoprotein in exosomes—was internalized by epithelial cells to induce EMT [106]. It is generally assumed that β-catenin will be degraded by ubiquitination and weaken Wnt signaling under normal conditions. Are exosomes necessary for the level of intracellular β-catenin? A novel study revealed that CD82 and CD9 contributed to the encapsulation of β-catenin in exosomes and its release into the extracellular environment, in which E-cadherin was essential for the secretion of β-catenin [107]. However, whether tumor cells suppress the release of exosomal β-catenin and thus enhance cellular motility remains to be explored.

Exosomes also contain matrix metalloproteinases (MMPs) which can degrade most protein components in the extracellular cell matrix (ECM), destroy the histological barrier of tumor cells’ invasion, and are involved in tumor metastasis [108,109]. Studies have shown that NPC-derived exosomes were able to activate AKT and ERK signaling pathways to enhance metastasis properties of tumor cells. High expression of MMP13 in exosomes secreted by NPCs cells was considered to play an important role in these processes. Exosomes released from MMP-13 silenced NPC cells reversed EMT compared with normal NPC cells, confirming the positive relationship between MMP-13 and EMT [110]. On the basis of this work, Shan et al. found that hypoxic conditions promoted the expression of MMP-13 in NPC cells, and more MMP13 was encapsulated in exosomes and delivered to recipient cells [111].

In some way, cytoskeleton proteins-rich exosomes initiated the metastasis cascade by inducing the translocation of adhesion molecules [112]. Cytoskeleton-associated proteins-enriched exosomes from metastatic colon cancer cells affected the integrity of endothelial cells by inducing a clear cytosolic delocalization of the adherens junction proteins including β-catenin, p120-catenin, and VE-cadherin, without changes of VE-cadherin expression at both mRNA and protein levels, through RhoA/Rock signaling in recipient cells. p53 deficient mouse bone marrow MSC (mBMMSC) exosomes enriched ubiquitin protein ligase E3 component N-recognin 2 (UBR2) resulted in the increased proliferation and migration of p53^+/+^ mBMMSC and murine foregastric carcinoma (MFC) by activating Wnt/β-catenin signaling with changed EMT marker expression including E-/N-cadherin [113]. In a separate study, the authors confirmed that many of the genes with human papilloma virus-16 (HPV-16) associated with oropharyngeal cancer (HPVOPC) had missense mutations and increased copy counts. After treatment with HPVOPC-derived exosomes, the EMT phenomenon, especially greater motility was observed in epithelial cells [114].

Notably, abnormal expression of soluble E-cadherin (sE-cadherin) in malignant ascites has been closely explored. sE-cadherin in exosomes derived from ovarian cancer cells was found to bind VE-cadherin to activate the β-catenin and NF-κB signaling pathways, and subsequently enhance angiogenesis in recipient cells [115]. It may be a novel regulatory mechanism for tumor angiogenesis.

## 6. Conclusions

In this review, we describe in detail how tumor-derived exosomes mediate the instability of cadherins and facilitate tumor progression. EMT occurs at the initial stage of tumor metastasis and is characterized by down-regulation of the epithelial marker E-cadherin and up-regulation of the mesenchymal marker N-cadherin. Abnormal expression of cadherins further results in the metastasis of tumor cells to distant organs or tissues. Conversely, delocalization of cadherins can also affect tumor cells to break through the endothelial barrier.

**Figure 3:**
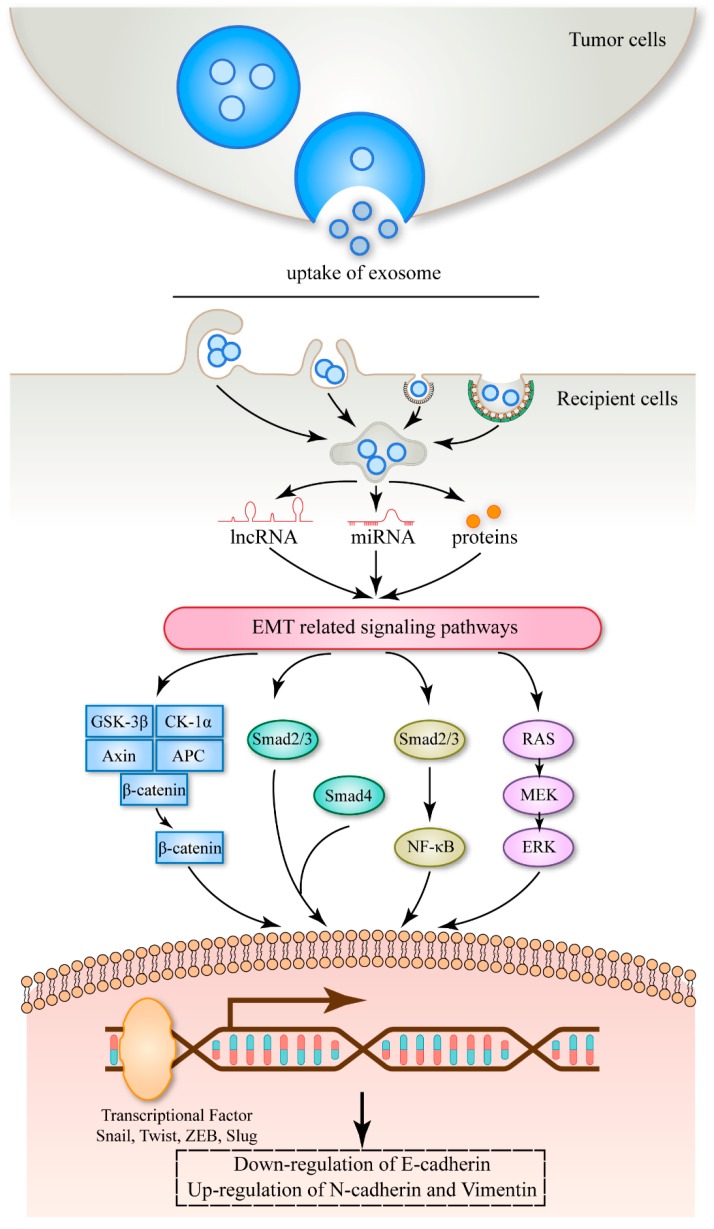
A schematic model of tumor-derived exosomes dysregulating cadherin expression. Tumor-derived exosomes deliver miRNA, lncRNA, and proteins to recipient cells, stimulate EMT-related signaling pathways, down-regulate the expression of E-cadherin, and up-regulate the expression of N-cadherin and vimentin, and further promote tumor progression.

Exosomes, a cargo of intercellular communication, are encapsulated with many bioactive substances. Usually, tumor-derived exosomes may carry more carcinogens or less tumor suppressors. Tumor-derived exosomes tend to transfer EMT-related RNAs and proteins to recipient cells, mediate the instability of cadherins, and promote cancer progression (Figure 3).

## Figures and Tables

**Table 1:** Summary of tumor-derived exosomal non-coding RNAs mediating the instability of cadherins.

Exosomal non-Coding RNAs	Cancer Types	Targets	Promote (✓)/Inhibit (✕) Tumor Progression
miR-9 [62]	Breast cancer	E-cadherin	✓
miR-103 [6]	Hepatocellular carcinoma	VE-cadherin	✓
miR-939 [63]	Breast cancer	VE-cadherin	✓
miR-148b [65]	Endometrial cancer	DNMT1	✕
miR-21 [68]	Oral squamous cell carcinoma	PTEN PDCD4	✓
miR-200c [72]	Colorectal cancer	ZEB1	✕
lncRNA-UCA1 [93]	Bladder cancer		✓
linc-ROR [94]	Thyroid cancer		✓
lncRNA-Sox2ot [99]	Pancreatic ductal adenocarcinoma	miR-200 family	✓
lnc-MMP2-2 [100]	Lung cancer	MMP-2	✓
lncRNA NONHSAT105177 [101]	Pancreatic ductal adenocarcinoma		✕
